# Patients’ socioeconomic status and their evaluations of primary care in Hong Kong

**DOI:** 10.1186/1472-6963-13-487

**Published:** 2013-11-25

**Authors:** Onikepe Owolabi, Zhenzhen Zhang, Xiaolin Wei, Nan Yang, Haitao Li, Samuel YS Wong, Martin CS Wong, Winnie Yip, Sian M Griffiths

**Affiliations:** 1 Faculty of Epidemiology and Population Health, London School of Hygiene and Tropical Medicine, Keppel Street, WC1E 7HT, London, UK; 2 School of Public Health and Primary Care, The Chinese University of Hong Kong, Hong Kong, SAR, PR China; 3 Department of Public Health, Oxfordshire Primary Care Trust, Richard Building, Old Road Campus, Headington, Oxford OX3 7LG, UK

**Keywords:** Primary care, Socioeconomic factors, PCAT, Hong Kong

## Abstract

**Background:**

Strengthening primary care is key to Hong Kong’s ongoing health system reform. Primary care remains unregulated, private sector dominated and financed mainly out-of-pocket. This study sought to examine the association between patients’ socioeconomic status (SES), source of health payments and the quality of primary care they accessed to inform policy discussions.

**Methods:**

Data was collected from 1,994 respondents in a stratified random telephone survey with a 68% response rate, using the validated primary care assessment tool (PCAT). Education, household-income and type of housing were selected as indicators of SES. Multivariable ordinal logistic regression models were created to examine associations between indicators of SES and scores of quality.

**Results:**

Higher household-income was most significantly associated with better experiences of quality. Respondents with HK$ 15000–39999 (USD1934-5158) and HK$ 40000 (USD5159) and above were 47% (OR 1.47, 95% CI 1.10-1.96) and 2 times (OR 2.07, 95% CI 1.38-3.09) more likely to experience better quality than the lowest-income group respectively. Income group HK$ 40000 (USD5159) and above was 84% more likely to have better utilization (OR 1.84, 95% CI (1.21-2.78), and 2 times more likely to receive better comprehensiveness (OR 1.90, 95% CI 1.26-2.87). Patients who used only private insurance were 80% (OR 1.80, 95% CI 1.20-2.68) more likely to experience better quality than those who paid out-of-pocket.

**Conclusions:**

Our results show that the quality of primary care experienced in HK tended to be higher for those who had higher income and private insurance, and were able to pay out-of-pocket for the care. This indicated that the inequality in primary care is likely to be related with the private dominated primary care system in Hong Kong. More public responsibility on primary health care should be sought for in HK and similar contexts to reduce the inequality in primary care.

## Background

Primary care is recognized internationally as the cornerstone of strong health systems [1] and strengthening primary care is a key element of many national health systems reform. Good primary care has been shown to mitigate socioeconomic disparities in healthcare utilization, is associated with better and more equitable health outcomes, and fosters greater patient satisfaction [1]. With increased patient participation in healthcare, patients’ assessments of quality and satisfaction have become important outcomes in evaluating health services [2-4].

Measuring quality of healthcare is complex. However, the attributes of primary care have been used to develop measurement scales by researchers [5]. These attributes have been found to be associated with quality of care [6,7] and are the most relevant characteristics of effective primary care organization and delivery at the population level. They are: 1) First contact access to care; 2) ongoing care; 3) Comprehensiveness of care; and 4) Coordination of care [1,8].

Hong Kong is a high-income, special administrative region of China, with a highly developed, mixed healthcare system, and population health indicators comparable to those in high-income western nations. Rapid health expenditure growth, and a fast aging population have stimulated consultations on health systems reform in Hong Kong since 1993 [9]. Strengthening the role of primary care is a key element of the ongoing reform strategy [9]. Although Hong Kong’s underlying ethical principle in health care is that “no one shall be denied healthcare due to a lack of means” [10], it is to all intents egalitarian on access to care not quality received. In practice, the private sector provides the largest proportion of primary care (70%) and operates under free market principles [11-13]. Physician reimbursement therein is usually fee for service (FFS), care is funded primarily by out-of-pocket (OOP) payments and charges are not subject to government regulation [14,15]. The exception to OOP payments is private insurance, which covers about 27% of the population [11] and is generally regressive as it is usually provided by employers and tied to staff seniority [13]. Insufficient regulation, has fostered marked variation in the quality available, poor coordination with other levels of care within the system, and substantial supplier-induced demand [15]. The other 30% of primary care is provided by 74 government-run general outpatient clinics (GOPC’s). GOPCs provide service to all the population, but target the poor and the elderly population. GOPCs are highly subsidized (over 90%) by the government, and are usually overcrowded. However, patient reported lower quality of care on accessibility and communication compared with private care clients [16]. In general, affordability is still said to be a major impediment to receiving primary care, and doctor shopping is prevalent amongst patients [17]. Policy analysis documents identify that primary care in Hong Kong is provider-dominated, and fragmented [18].

Our study is part of a larger research project called - “Using a systematic approach to evaluate primary care development in Hong Kong, Shenzhen, Kunming and Shanghai”. The overall study aims to measure the impact of healthcare reform policies on structure, process, output and outcome of primary care in the four cities. Wong et al.’s 2010 study [19] on the quality of primary care compared quality between the public and private sector. No studies have explored the inequity in the quality of primary care utilized by different socioeconomic groups of patients yet. Consequently, our study examined the association between patients’ socioeconomic status, and experiences of primary care. This should provide useful information for ongoing policy discussions about primary care organization and delivery.

## Methods

### Measurement tool

#### The primary care assessment tool (PCAT)

Our study used the Primary care Assessment Tool-Adult short version (PCAT-AS) for data collection. It was developed by Starfield and Shi at the John’s Hopkins primary care policy center [6,7]. The patient tool assesses patients experience of primary care rather than their satisfaction and thus attempts to capture quality based on the characteristics outlined by the World Health Organization and Institute Of Medicine definitions [19]. Haggerty et al’s 2011 study has showed that the PCAT have satisfactory validity and reliability [20]. It evaluates a wide breadth of attributes and its scales are specific to the core primary care attributes. Hence, it is easily interpreted from a policy standpoint. Our tool has been translated into Cantonese Chinese from English, validated [21] and used in Hong Kong in a previous study [19]. The primary care provider within this tool was defined using the 3 questions asked in the original PCAT survey- “is there a doctor that you usually go to if you’re sick and need advice about your health?” “Is there a doctor that knows you best as a person?” and “Is there a doctor or place that is most responsible for your health care?” We examined data to see what proportion of respondents had the same provider for 2 or 3 of these questions to understand how clearly they defined their primary care provider. For subsequent interview questions, when all 3 providers were the same, that provider was used as the primary care provider. If the response to “the usual doctor” was the same as for either of the other 2 questions, then the “the usual doctor” provider was used. However, if the response for “the usual doctor” was different, but the responses to the 2 other questions were the same, then the provider where both are the same was used. Finally, if all 3 responses were different, then the provider identified for “the usual doctor” was used [22]. The PCAT measures the four core domains of primary care i.e., first contact, ongoing care, coordination, and comprehensiveness (see the Definition of the domains of the primary care assessment tool subsection), divided into 8 core subdomains [23] Specific definitions of each subdomain is published in Wong’s study [19], which consists of 3–15 questions. Answers are ranked on a 4-point Likert-type scale with “1” indicating ‘Definitely Not’ , “2” ‘Probably Not’ , “3” ‘Probably’ , and “4” ‘Definitely’. The total score for each subdomain was calculated by summing the values of all items under it with reverse coding conducted where appropriate. Missing items or non-response was managed according to the scoring instructions provided with the tool. The overall primary care score (total PCAT score) of quality was calculated by adding the mean scores of each of the 8 core subdomains [23]. In this study, 4 out of the 8 subdomains reflecting the process of primary health care delivery and the total primary care assessment tool score were used as outcome variables. The four subdomains are first contact utilization, ongoing care, coordination and comprehensiveness provided.

### Definition of the domains of the primary care assessment tool

•**First contact access to care**: the first contact with the health care system with services that are accessible and at a close proximity

•**Continuity of care**: interventions cover the patient’s health needs throughout the course of their lives

•**Comprehensiveness of care**: providing care for common problems including providing curative, rehabilitative and supportive care, as well as health promotion and disease prevention

•**Coordination of care**: seamless care so that when patients are referred elsewhere the advice they receive is integrated into their care.

The patient tool collects additional individual information including household-income; insurance coverage; education; geographical districts; age; self-reported health status; and gender. These were adapted to suit Hong Kong’s context.

### Data collection

A stratified random telephone survey using the PCAT tool was conducted with Cantonese Chinese-speaking residents of Hong Kong aged 18 and above by trained interviewers from the Telephone Survey and Research Design Laboratory in Hong Kong. Survey interviews were conducted in the last quarter of 2011. Three major geographic regions were stratified and 16,662 telephone numbers were randomly selected from a telephone directory. No interviews were attempted in non-Cantonese households (about 1% in Hong Kong), commercial numbers, or fax numbers. We aimed to obtain 2000 completed surveys to detect a mean difference in the overall primary care score of 0.5 between the users of public and private providers, at an alpha level of 0.005, and power of 80% allowing for a 70% response rate. This was estimated based on a previous study in Hong Kong [19].

**Figure 1:**
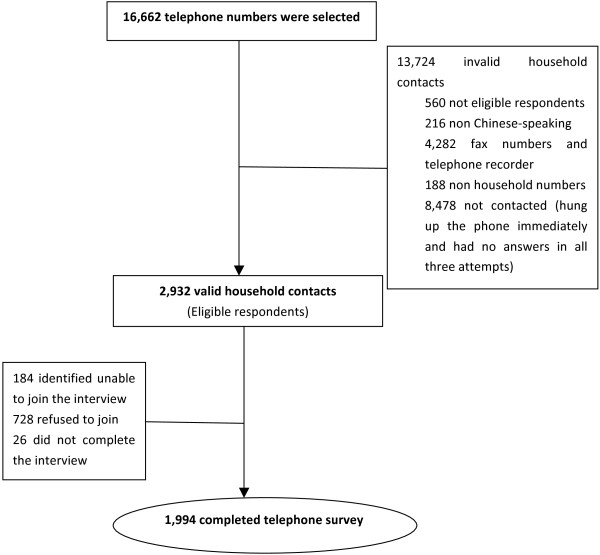
Schematic diagram showing the selection of participants for the study.

The details of participant selection are shown in Figure 1. A total of 2,932 respondents were eligible out of which 1994 completed the interview. The overall response rate (number of completed interviews divided by the total number of valid contacts) was 68%.

### Statistical analysis

Socioeconomic status of subjects was described using three indicators: household-income, education and type of housing. The median monthly domestic household income in Hong Kong in 2011 was HK$ 20,200 (USD2605). Monthly household income was categorized into 3 groups based on the 2011 Thematic Report regarding household income distribution in Hong Kong [24]: below HK$ 15,000(USD1934), between HK$ 15,000 and 39,999 (USD1934-5158), and HK$ 40,000 (USD5159) and above. Source of health payments were divided into 4 groups: only out of pocket payments, only the medical waiver scheme, only private insurance, and any combination of sources which also included people using medical waivers. Scores of the four main domains of primary care and the total primary care assessment score were calculated.

Multivariable ordinal logistic regression was conducted to examine the association between indicators of socioeconomic status and the primary care assessment score. Thereafter, we attempted to disentangle the components of the total primary care score by using the 4 subdomains representing the fulfillment of the attributes of primary care as outcome variables (first contact utilization, ongoing care, coordination information systems and comprehensiveness provided) [23]. In the multivariable model, all covariates were entered simultaneously. We controlled for type of healthcare provider, indicators of health need and demographic characteristics. All analyses were conducted using STATA version 12.1 (Statacorp).

## Results

The demographic characteristics of our respondents were compared with the general population. The age distribution of our survey respondents did not differ significantly from the general population (2011 Hong Kong census data, p = 0.62). Our respondents were however more likely to have higher education than the general population (p < 0.001). Table 1 shows the socioeconomic, demographic and healthcare service characteristics of our respondents. Majority of respondents (74.5%) named a private provider as their primary care source, and financed their care in the past 12 months by only out of pocket payments (68.4%). Majority of the respondents (82%) had the same provider for at least 2 of the questions to define primary care while 79% had the same provider for all 3 questions.

**Table 1:** Summary of the characteristics of the study population

**Variable**		**Frequency (%)**
*Individual characteristics*		
Gender	Male	938 (47.0%)
	Female	1056 (53.0%)
Age group (years)*	15-24	268 (13.5%)
	25-34	329 (16.5%)
	35-44	383 (19.3%)
	45-54	416 (20.9%)
	55-64	291 (14.6%)
	Above 65	303 (15.2%)
*Socioeconomic indicators*		
Education*	Not educated or preschool	97 (4.9%)
	Primary school	288 (14.7%)
	Secondary school	947 (48.2%)
	Above secondary school	632 (32.2%)
	Other	1 (0.05%)
Household income (HK$)*	Below 15000 (<USD1934)	449 (33.5%)
	15,000-39999 (USD1934-5158)	652 (48.7%)
	40000 and above (≥USD5159)	239 (17.8%)
Area of residence*	Hong Kong island	423 (21.4%)
	Kowloon	535 (27.1%)
	New territories	1015 (51.4%)
Type of housing*	Public rental housing	595 (31.5%)
	Private permanent housing	1285 (68.0%)
	Others	9 (0.5%)
*Health financing*		
Source of health payments in the past 12 months*	Out of pocket only	1297 (67.1%)
	Multiple sources	413 (21.3%)
	Medical waiver scheme only	66 (3.4%)
	Private insurance only	159 (8.2%)
*Source of healthcare*		
Usual primary care provider*	Government clinic	390 (21.2%)
	Private provider	1370 (74.5%)
	Traditional Chinese practitioner	78 (4.2%)
*Need related variables*		
Self-rated health*	Poor	726 (36.5%)
	Good	1262 (63.5%)
Chronic disease status	No chronic disease	1439 (72.2%)
	Any chronic disease	555 (27.8%)
*Satisfaction*		
How acceptable is the amount you pay for health care?*	Not acceptable	267 (13.4%)
	Acceptable	1720 (86.6%)

**Note:** *Indicates that this variable has missing observations. Total number of cases in dataset is 1994.

Conversion rate is USD ($)1.00 = HK$7.75.

Table 2 presents the results of the comparison of PCAT scores among participants with different household incomes and sources of payments for healthcare in the past 12 months. It shows that the total PCAT score increases within each higher income category. Notably, respondents who have household-income lower than HK$ 15,000(USD1934) reported significantly poorer experiences in all domains except for first contact utilization. Respondents who used only private insurance had significantly higher total PCAT score compared with other sources of health payments.

**Table 2:** Comparison of total PCAT scores among patients with different household incomes and sources of payments for healthcare

	**Total PCAT score**			**4 Primary care subdomains**							
**Variable**	**Range of values**	**Mean (SE)**	***p *** **value**	**First contact utilization domain: mean (SE)**	***p *** **value**	**Ongoing care domain: mean (SE)**	***p *** **value**	**Co-ordination information systems mean (SE)**	***p *** **value**	**Comprehensiveness provided domain: mean (SE)**	***p *** **value**
**Self-reported household income**											
<HK$ 15000 (<USD 1934)	8.63-28.2	18.77 (3.12)	0.0009	3.04 (0.78)	0.38	2.69 (0.63)	<0.001	2.74 (0.54)	0.0004	1.99 (0.66)	0.035
HK$ 15,000- 39999 (USD 1934- 5158)	8.25-29.73	19.25 (3.23)		3.08 (0.81)		2.92 (0.65)		2.79 (0.54)		2.07 (0.64)	
≥HK$ 40000 (≥USD 5159)	9.17-30	19.73 (3.75)		3.13 (0.87)		3.03 (0.61)		2.91 (0.51)		2.13 (0.64)	
**Sources of health payments in the past 12 months**^**a**^											
Out of pocket only	7.2-30	18.98 (3.37)	0.0004	3.02 (0.85)	0.053	2.88 (0.65)	<0.001	2.74 (0.55)	0.29	2.04 (0.68)	0.29
Medical waiver only	8.5-26.73	18.52 (4.33)		3.16 (1.02)		2.3 (0.71)		2.76 (0.65)		1.98 (0.9)	
Private insurance only	10.23-27.15	20.12 (3.53)		3.19 (0.88)		3.03 (0.72)		2.8 (0.49)		1.98 (0.78)	
Multiple sources	10-29.9	18.91 (3.29)		3.02 (0.76)		2.86 (0.64)		2.79 (0.55)		2.09 (0.62)	

**Note**: ^a^Indicates that this variable has missing observations. Total number of cases in dataset is 1994.

Conversion rate is USD ($)1.00 = HK$7.75. http://www.oanda.com, 14^th^ October 2013.

Table 3 shows the adjusted odds ratios for our regression models where the total PCAT score is the dependent variable. Individuals with household-income between HK$ 15000-39999 (USD1934-5158) and HK$ 40000 (USD5159) and above are 47% (OR 1.47, 95% CI 1.10-1.96) and 200% (OR 2.07, 95% CI 1.38-3.09) more likely to have higher total PCAT score than the lowest income group respectively. Type of provider was also significant but in 2 different directions. Individuals using private care are 200% more likely to have higher a total PCAT score (OR 2.07, CI 1.53-2.82) compared with patients using government clinics. However, those using traditional Chinese Medicine are 63% less likely to have a good total PCAT score (OR 0.37, CI 0.19-0.70). There is a significant association between how health care was financed and primary care experience. Compared with individuals using only OOP, those who used only private insurance were 80% (OR 1.80, 95% CI 1.20-2.68) more likely to have a higher total PCAT score. Similar results were obtained when OLS linear regression was used as a sensitivity analysis.

**Table 3:** Multivariable ordinal logistic regressions showing: The association between scores of primary care and the 4 core primary care domains and explanatory variables

	**Dependent variables OR (95% C.I)**				
**Independent variables**	**Total PCAT score**	**First contact utilization domain**	**Ongoing care domain**	**Coordination (information systems)**	**Comprehensiveness provided domain**
	**(N = 1,159)**	**(N = 1,149)**	**(N = 1,155)**	**(N = 1,157)**	**(N = 1,156)**
*Socioeconomic indicators*					
**Household income**					
<HK$ 15000 (<USD1934)	1.0	1.0	1.0	1.0	1.0
HK$ 15,000- 39999 (USD 1934–5158)	1.47 (1.10-1.96)**	1.33 (1.00-1.79)*	1.07 (0.78-1.46)	1.18 (0.87-1.61)	1.46 (1.07-1.93)*
≥HK$ 40000 (≥USD5159)	2.07 (1.38-3.09)***	1.84 (1.21-2.78)**	1.27 (0.82-1.97)	1.61 (1.04-2.47)*	1.90 (1.26-2.87)**
**Education**					
Secondary and below	1.0	1.0	1.0	1.0	1.0
Above secondary	1.13 (0.87-1.49)	0.91 (0.69-1.20)	1.07 (0.80-1.44)	1.23 (0.92-1.64)	0.93 (0.71-1.23)
**Type of housing**					
Public rental housing	1.0	1.0	1.0	1.0	1.0
Private permanent housing	1.11 (0.86-1.45)	1.06 (0.81-1.38)	1.24 (0.93-1.64)	0.89 (0.67-1.17)	0.98 (0.75-1.28)
Others	0.67 (0.15-2.86)	1.03 (023–4.71)	1.26 (0.26-6.19)	1.21 (0.26-5.60)	1.21 (0.26-5.77)
*Health financing*					
**Source of health payments in the past 12 months**					
Out of pocket only	1.0	1.0	1.0	1.0	1.0
Multiple sources	0.78 (0.60-1.01)	0.82 (0.63-1.08)	1.03 (0.79-1.41)	1.15 (0.83-1.48)	0.96 (0.73-1.26)
Medical Waiver scheme only	1.15 (0.51-2.55)	1.43 (0.64-3.17)	1.63 (0.73-3.62)	1.32 (0.56-2.96)	0.81 (0.36-1.82)
Private insurance only	1.80 (1.20-2.68)**	1.67 (1.09-2.56)*	1.51 (0.97-2.36)	0.89 (0.59-1.40)	0.52 (0.34-0.80)**
*Source of healthcare*					
**Usual primary care provider**					
Government clinic	1.0	1.0	1.0	1.0	1.0
Private provider	2.07 (1.53-2.82)***	0.88 (0.65-1.21)	11.76 (8.19-16.89)***	0.88 (0.66-1.22)	1.07 (0.78-1.47)
Traditional Chinese practitioner	0.37 (0.19-0.70)**	0.06 (0.03-0.13)***	34.99 (17.45-70.16)***	0.26 (0.13-0.52)***	2.66 (1.39-5.09)**
*Need related variables*					
**Self-rated health**					
Poor	1.0	1.0	1.0	1.0	1.0
Good	1.39 (1.07-1.82)*	1.54 (1.17-2.02)**	1.30 (0.99-1.84)	1.00 (0.76-1.33)	1.31(1.00-1.72)
**Chronic disease status**					
No chronic disease	1.0	1.0	1.0	1.0	1.0
Any chronic disease	3.17 (2.37-4.25)***	1.95 (1.46-2.62)***	1.35 (0.99-1.84)	1.34 (0.99-1.82)	1.57 (1.17-2.10)**
*Individual characteristics*					
**Gender**					
Female	1.0	1.0	1.0	1.0	1.0
Male	0.78 (0.63-0.97)*	0.97 (0.77-1.22)	0.87 (0.68-1.11)	0.97 (0.76-1.22)	1.03 (0.82-1.29)
**Age group (years)**					
15-24	1.0	1.0	1.0	1.0	1.0
24-64	0.89 (0.61-1.30)	0.89 (0.59-1.33)	0.86 (0.54-1.27)	1.59 (1.06-2.39)*	0.78 (0.52-1.17)
Above 65	1.02 (0.61-1.73)	0.81 (0.47-1.40)	0.70 (0.39-1.24)	0.84 (0.48-1.45)	0.79 (0.46-1.37)
*Satisfaction*					
**How acceptable is the amount you pay for health care?**					
Not acceptable	1.0	1.0	1.0	1.0	1.0
Acceptable	1.35 (0.98-1.86)	1.01 (0.73-1.41)	1.88 (1.30-2.70)***	1.11 (0.78-1.58)	1.03 (0.74-1.43)

**Note:** ***p-va1ue < 0.001, **p-value < 0.01, *p-value < 0.05.

Conversion rate is USD ($)1.00 = HK$7.75. http://www.oanda.com, 14^th^ October 2013.

Further analysis attempted to disentangle the overall experience of primary care quality by examining the association between patient’s experiences of each core attribute of primary care and the explanatory variables. Table 3 shows the adjusted odds ratios from multivariable regressions where we fit the same model for 4 domains: first contact utilization, ongoing care, coordination information and comprehensiveness provided, as the dependent variables. Notably, household-income is the only indicator of SES to show an association with the primary care attributes. Respondents with household-income, respondents between HK$ 15000-39999 (USD1934-5158), and HK$ 40000 (USD5159) and above, are 33% (OR 1.33, 95% CI 1.00-1.79) and 84% (OR 1.84, 95% CI 1.21-2.78) respectively more likely to have higher first contact utilization compared with the lowest group. Similarly, respondents with household-income HK$ 40000(USD5159) and above are 61% (OR 1.61, 95% CI 1.04-2.47) more likely to experience better coordination compared with the lowest income group. The association with comprehensiveness increases within each higher income category. As seen in Table 3, persons in HK$ 15000-39999(USD1934-5158), and in HK$ 40000 (USD5159) and above are 46% (OR 1.46, 95% CI 1.07-1.93) and 90% (OR 1.90, 95% CI 1.26-2.87) respectively more likely to experience more comprehensiveness than those with household-income below HK$ 15000 (USD1934).

Health payment source shows significant associations with first contact utilization, and comprehensiveness provided. Compared with respondents who pay only OOP, people who have private insurance are 67% more likely to have high first contact utilization (OR 1.67, 95% CI 1.09-2.56). However, they are 48% (OR 0.52, 95% CI 0.34-0.80) less likely to experience good comprehensiveness.

The type of healthcare provider shows a very significant relationship with ongoing care. Respondents who reported private providers and traditional Chinese practitioners had a 12 fold (OR 11.76, 95% CI 8.19-16.89), and 35 fold (OR 34.99, 95% CI 17.45-70.16) odds respectively of a more longitudinal relationship compared with respondents using government clinics.

## Discussion

We examined socioeconomic differences in patients’ evaluations of primary care quality, using a validated tool in Hong Kong. Consistent with other studies [9,19], majority of our respondents named a private provider as their primary care source, and financed their care in the past 12 months by only out of pocket payments. Our study shows that people with higher income experienced significantly better overall primary care quality compared with people with the lowest income. There appears to be a trend of higher quality rating as income increased across the three groups. Similarly compared with individuals who only paid OOP for care, patients who used only private insurance had significantly higher odds of good quality experience. People utilizing private care also had better quality experiences compared with those using government clinics.

A US based study showed that people with low socioeconomic status had worse ratings of their healthcare provider [25], Conversely a 2001 UK study showed that socioeconomic differences accounted for a small amount of variability in patient evaluations of care [26], One previous study in Hong Kong suggested that the public specialist outpatient care was highly equitable [18]. However Wong et al’s study suggested the quality experienced by patients differed based on the provider. Patients using private providers had significantly higher odds of better quality primary care than GOPC patients [19]. These findings are similar to those from developing countries with a large for-profit private sector and high out-of-pocket payments, wherein quality of service tends to vary by cost [27]. In most of Europe source of health payments doesn’t typically account for variability in quality scores, because of the universal coverage, standardized quality of primary care and prepaid financing studies. However evidence from the US [28] and Hong Kong [19] has demonstrated an association between prepaid care and higher quality experiences. This contrast in how SES affects patient evaluations may be due to differences in organization, financing and delivery of primary care in both contexts [29]. Similar to Hong Kong, the US has a largely provider driven, private and fragmented primary care sector in contrast to the UK which has publicly delivered primary care with a gate-keeping function and no payments demanded at point-of-care [26].

This study found a strong association between having a poorer quality primary care experience, and being in a lower-income group in Hong Kong. One possible explanation is that choice of provider is dictated by patients’ ability to pay. Previous work has shown that the cheaper it is, the higher the chance it is of lower quality [30]. It may also be that providers tend to deliver inferior care to patients with lower SES due to reduced ability to pay [31]. Exploring the diversity within private care, which is the most frequent source of care, may provide more insight into the association between income and quality experienced. We also found an association between how patients paid for their healthcare and the quality they experienced. Private insurance is predominant amongst the higher cadre of the workforce [9], thus such people may well be able to access higher quality care generally. Alternatively, the coverage afforded by prepayment may allow for consistent receipt of more services or higher quality services, for people that have insurance even within the middle-income group.

Further analysis examined the association between quality experienced for each attribute of primary care and explanatory variables. Higher Household-income is significantly associated with higher first contact utilization and comprehensiveness scores. This is consistent with studies showing that people with higher incomes have higher utilization of primary care in Hong Kong controlling for differences in need [32,33]. In contrast, international experience from other high-income OECD countries except the US usually shows that primary care utilization is pro-poor [34,35]. Primary care utilization in Hong Kong is largely funded OOP thus cost may be a major deterrent for lower-income people [36]. While private insurance also showed significant association with higher first contact utilization compared to out-of-pocket payments, it was associated with lower comprehensiveness. This may be associated with available packages and requires further research.

Our results also suggested that “acceptability of health expenditure”, which was used as a measure of satisfaction, was significantly associated with better ongoing care but not with any other domain. This correlation is consistent other research showing that that price and patients satisfaction affects patient’s decisions to continue with the same provider [37]. It is also consistent with the studies in Hong Kong showing that dissatisfaction with cost is the commonest cause of doctor shopping, which compromises the continuity of primary health care [17,38].

Overall healthcare financing in Hong Kong is progressive since it is tax based [33]. However, out-of-pocket payments for primary care make access and thus utilization biased towards people with higher-incomes. In the short term the government should realign financial and incentives to help improve utilization by patients and comprehensiveness of services provided. Possible options include: encouraging private insurance providers to provide better coverage for primary care [36], and standardizing charges for private healthcare. Similarly scaling up the reform strategy to create primary care networks and facilitate access to electronic care records may greatly improve coordination [10]. With its health reform goals already in place, taking account of inequities should be a priority in monitoring plans, such that policy and program design is aligned to achieve the desired goal of an equitable and financially sustainable health system.

### Limitations

Several limitations were identified in our study. First, the use of a telephone survey may limit its representativeness. The response rate of 68% to the survey may limit the generalizability of results to only respondents, as their characteristics, health-seeking behavior and perceptions of quality may be different from those who refused to be interviewed. Second, the PCAT-AS tool algorithm for identifying the primary care provider has scope for people to speak about a usual source of care, which might be a specialist consulted frequently for a specific health problem rather than a primary care provider. These 2 categories of respondents e.g. those with a primary care provider versus those who speak about a usual source of care who might be a specialist may differ considerably, and comparing them may be misleading. Third, data from this study were cross-sectional, which does not allow for demonstration of causality. Additionally all explanatory variables were self-reported and unverified. They are thus subject to recall and misclassification bias. We were unable to adjust for the effect of household family size on income, marital status of the respondent and clustering in primary care practice, as data on specific providers was not available in the dataset. Additionally an ordinal logistic regression was fitted to the data instead of an ideal multi level model of patient within providers practice.

Despite the above limitations, the findings of this study are consistent with the literature on Hong Kong and inequities in health systems in general. In particular, the association between income and sources of health payments with patient evaluations of quality cannot be attributed to bias or confounding. Additionally the PCAT-AS is a validated tool regarding actual patient experiences.

## Conclusions

In summary, we have shown that quality of care in Hong Kong appears to be associated with household income and how patients pay. Unlike most high-income countries wherein primary care utilization is pro-poor, it appears to be pro-rich in Hong Kong, largely due to incoherent financing and a provider-dominated health system. This indicated that the inequality in primary care is likely to be related with the private dominated primary care system in Hong Kong. More public responsibility on primary health care should be advocated for in Hong Kong and similar contexts to reduce the inequality in primary care.

## Competing interests

The authors declare that they have no competing interests.

## Authors’ contributions

XLW, SW, MW and SG designed the study. ZZZ, XLW, NY, HTL participated in data acquisition. OO and ZZZ are responsible for data analysis and interpretation. OO and ZZZ drafted the manuscript. XLW, SW, MW, WY and SG revised the draft for intellectual content. All authors helped in final approval of the completed article.

## Pre-publication history

The pre-publication history for this paper can be accessed here:

http://www.biomedcentral.com/1472-6963/13/487/prepub
